# Energy intake, expenditure and balance, and factors associated with energy balance of young adults (20–39 years): a retrospective cross-sectional community-based cohort study

**DOI:** 10.1186/s40795-022-00628-2

**Published:** 2022-12-05

**Authors:** Rufina N. B. Ayogu, Hannah Oshomegie, Elizabeth A. Udenta

**Affiliations:** Department of Nutrition and Dietetics, Faculty of Agriculture, University of Nigeria, Nsukka, Nigeria

**Keywords:** Energy intake, Energy expenditure, Energy balance, Associated factors, Young adults, Enugu State-Nigeria

## Abstract

**Background:**

Obesity epidemic presents major challenge to chronic disease prevention. Young adults may be at higher risk due to consumption of energy dense foods/beverages and low physical activity. This study assessed the energy intake, expenditure and balance of 20–39 year-old adults and also evaluated factors associated with energy balance to provide basis for obesity prevention and control.

**Methods:**

This retrospective cross-sectional cohort study involved 240 respondents selected through multistage random sampling. Data were collected through questionnaire, anthropometry and three 24-h dietary recall. Energy expenditure was assessed as the sum of resting energy expenditure (REE), energy expenditure of activity (EEA) and diet-induced energy expenditure of three days. Frequencies and percentages were employed in analysing general characteristics of the respondents. T-test and analysis of variance were used to assess relationships between and among numerical variables while relationships among categorical variables were evaluated through Chi-square test. Binary and multivariate logistic regression analyses were used to evaluate the factors associated with energy balance.

**Results:**

Majority (53.2%) had normal body mass index and this decreased as age increased (*P* < 0.001); 23.8% had overweight, 21.7% had general obesity, 38.3% had abdominal obesity and 39.2% had high risk waist hip ratio. Total energy intake (TEI) of the respondents was 2416.0 ± 722.728 kcal/day. Males had lower energy intake (kcal/day) of 2398.8 ± 494.761 than females (2431.1 ± 876.664). Male intake contributed less (85.7%) than female intake (110.5%) to recommended energy intake. TEI (kcal/day) was lowest among 25–29 (2285.3 ± 497.793) but highest (2586.0 ± 467.587) among 35–39 year-olds (*P* < 0.05). Total energy expenditure (TEE) was 2195.5 ± 384.544 kcal/day; a value of 2274.3 ± 385.792 was found among males while the females had a lower value of 2126.6 ± 371.403. TEE (kcal/day) ranged from 2169.4 ± 411.625 in 20–24 to 2248.8 ± 273.534 in 30–34 year-olds. Those with general obesity had higher energy (kcal/day) intake (2733.7 ± 1171.073), expenditure (2431.7 ± 491.666) and balance (302.0 ± 1300.186) than those without obesity (*P* < 0.01). Energy intake (2573.0 ± 966.553) and expenditure (2226.4 ± 372.621) were significantly higher among those with abdominal obesity than those with normal waist circumference (*P* < 0.05). Overall positive energy balance of the adults was 220.5 ± 787.271. The females had higher positive energy balance (304.4 ± 921.041) than males (124.5 ± 588.637). Energy balance was lowest (102.5 ± 629.780) among 25–29 and highest (373.5 ± 494.546) in 35–39 year-olds. Most (68.8%) of the participants had positive energy balance. Age (AOR:2.89, 95% C.I.:1.27–6.56) and occupation (AOR:2.30, 95% C.I.:1.05–5.03) increased the likelihood of positive energy balance by 2.

**Conclusions:**

The study showed lower energy intake among the respondents than the recommended value; females had a value higher than what was recommended, males had less. Majority had positive energy balance and this was mostly found among those with obesity. Age and occupation were factors associated with positive energy balance. Nutrition education, health education and dietary counselling are recommended strategies to control sustained weight gain.

## Background

Energy intake (EI) that exceeds energy expenditure is the main driver of weight gain. Energy consumed in foods are transformed to substrates that are either oxidized to produce metabolically useful energy that drives biological processes or stored [1] as fat when in excess. World Health Organization [2] reported that the principal reason for the problem of excess weight is a sustained energy imbalance between calories consumed and calories expended and numerous genetic and environmental factors play intermediary roles in this process. Food environment, marketing of unhealthy foods, urbanization and reduction in physical activity also play important roles [3].

Energy intake is dependent on carbohydrate (4 kcal/g, 17 kJ/g), protein (4 kcal/g, 17 kJ/g), and fat (9 kcal/g, 38 kJ/g) and can be assessed through dietary methods (weighed food intake, laboratory analysis of foods, quantified 24-h dietary recall and food frequency questionnaire). Expended energy reflects fuels metabolized for growth, body maintenance, physical activity, pregnancy, lactation and many other processes and the rate of whole-body energy expenditure varies within a 24-h period and across life span [1]. Total energy expenditure (TEE) is composed of energy costs of processes essential for life (basal/resting energy expenditure), energy expended during physical activities (energy expenditure of activity) and energy cost of digesting, absorbing and metabolising food (diet-induced energy expenditure) [4].

Obesity, a disease of excess body fat is the driver of non-communicable diseases such as cardiovascular diseases, musculoskeletal disorders and some cancers and has been linked to more deaths worldwide than underweight with the risk increasing as BMI increases [5]. Its prevalence has increased substantially across the globe with most evidence coming from high income countries and more research required in low- and middle- income countries [3]. In Nigeria, obesity prevalence has been reported as 8.1–22.2% [6], 17.0% [7], 13.1% [8] and 14.3% with higher prevalence among women and urban dwellers [9]. Simmond et al. [10] found obese children and adolescents to be about five times more likely to be obese in adulthood than those who were not obese implying that childhood obesity tracks into adulthood. The epidemic of overweight and obesity presents a major challenge to chronic disease prevention and health across the life cycle [11]. 

The risk for adult obesity may still be higher among young adults in urban areas as a result of excess energy intake mediated upon by rapid urbanization, change in food environment and consumption of energy dense foods and beverages, low physical activity, improved socio-economic status and means of transportation. Few studies have been conducted on energy intake and expenditure of young adults and to the best of our knowledge, none has been conducted in the study area. Based on this, this study aimed to assess the energy intake, energy expenditure and energy balance of young adults (20–39 years) in Nsukka urban and factors associated with their energy balance. Data generated from this study will facilitate interventions to reduce the prevalence and complications of obesity.

## Methods

### Study setting

The study was conducted in Nsukka urban. Nsukka is located in the northern part of Enugu State, Southeast, Nigeria with a total population of 309,633 people as at 2006 national census increasing at an annual rate of 3.0%. Major occupation includes farming, trading and civil service. Major crops and livestock consumed are cassava, yam, maize, cocoyam, rice and sweet potato, poultry, pigs, goats and sheep.

### Study design and participants

The study employed retrospective cross-sectional cohort design in the study of energy status and factors associated with energy balance of young adults (20–39 years). The study population comprised of all free living non pregnant non lactating young adults (20–39 years) in Nsukka urban. Those who refused to be included by not signing informed consent or unable to supply data for three consecutive days were also excluded.

### Sample size calculation

Sample size for the study was calculated using modified Cochran’s formula: *N* = 4P (1-P)/W^2^. Margin of error (5%), non-response rate (5%) and p value of 17.0% which is the prevalence of obesity among urban Nigerian adults was used to obtain a sample size of 240 [7].

### Sampling technique

A multi-stage probability sampling technique was used in selecting the respondents. In stage one, two (2) wards (Ihe and Mkpunano) out of 4 wards that make up Nsukka urban were selected using simple random sampling technique by balloting without replacement. In the second stage, one community (Onuiyi from Ihe and Umuakashi from Mkpunano) was selected from each ward by simple random sampling. In stage three, urban settlements (Onuiyi from Onuiyi and Army Barracks from Umuakashi) in the two communities were identified and included (on the basis of population density and ease of access to transport). Stage four involved systematic random selection of every 5^th^ living house in the area. Probability proportional to size was adopted. In the fifth stage, one household was selected from each house by simple random sampling technique. In the sixth and final stage, only two young adults within the ages of 20–39 years were selected from each selected household by simple random sampling using balloting without replacement. Where there was only one eligible adult, a second household was selected from the same house and if there was none, the next house was selected and stages five and six repeated.

### Ethical clearance and consent to participate

Ethical approval for the study was obtained from Health Research Ethical Committee, University of Nigeria Teaching Hospital (UNTH) Ituku-Ozalla, Enugu State (NHREC/05/01/2008B-FWA00002458-1RB00002323). After details of the study were explained to them, respondents were requested to sign an informed consent form indicating their willingness to participate in the study.

### Data collection methods

A validated questionnaire was used to obtain data on socio-demographic, dietary habits and lifestyle characteristics of respondents. WHO global physical activity questionnaire administered by trained interviewers was used to assess physical activity level of the respondents.

Weight was measured to the nearest 0.1 kg with 120 kg capacity Hanson’s bathroom weighing scale. Participants stood erect in minimal clothing with arms hanging by the sides and no shoes on. Height (in cm) was taken with height meter rule with bare feet parallel to each other and heels, buttocks, shoulders and back of head touching the height meter rule. Body mass index (BMI, kg/m^2^) derived as weight to height ratio (weight in kg/height in metre squared) was used to classify subjects into underweight (< 18.5), normal weight (18.5 – 24.9), overweight (25.0 – 29.9) and obese (> 30). Waist circumference (WC) in centimetres was measured at the end of expiration using a flexible, non-stretchable tape placed at the midpoint between the top of the iliac crest and lower margin of the last palpable rib while participants stood upright. Hip circumference (in cm) was measured around the widest portion of the buttocks. Ratio of waist to hip circumference (WHR) was calculated. WC > 94 cm in males and > 80 cm in females were taken as abdominal obesity; WHR > 0.85 for females and > 0.90 for males were considered high (health) risks [12].

Three 24-h dietary recall involving two weekdays and one weekend day and a total of 6 meals per day was conducted by trained interviewers to determine the energy intake of the respondents [13, 14]. Respondents were requested to describe the types, brand names and quantity of ingredients, method of preparation/cooking and portion sizes of foods (meals, snacks and beverages/drinks) consumed during the period under study whether at home or outside the home. Quantification of the reported foods and beverages/drinks was achieved with weights and volumes of household measures (cups, glasses, bowls, jugs, spoons, plates, slices) and food items/models of different sizes. Estimated amounts were weighed using kitchen scales and the results recorded in grams. Macronutrient (protein, carbohydrate and fat) values of the foods were obtained from West African and Nigerian food composition tables and results of food/diet analysis reported in journal articles [15–18]. These were used to estimate the energy values of each food/snack and beverage/drink consumed based on Atwater factor of 4, 4 and 9 kcal/g for protein, carbohydrate and fat, respectively. The values for the three days were summed up and divided by three to obtain the mean daily energy intake. The mean values were used in statistical analysis.

Total energy expenditure (TEE) was determined as the sum of resting energy expenditure (REE), energy expenditure of activity (EEA) and diet-induced energy expenditure (DEE) based on three days’ assessment. Mean of the total energy expenditure (kcal/day) for the three days was used in statistical analysis.

(A) Resting energy expenditure (kcal/day) was obtained through Harris-Benedict’s predictive equation [14, 19] for males (66.5 + (13.75 × weight in kg) + (5.003 × height in cm) – (6.75 × age in years)) and females (655.1 + (9.563 × weight in kg) + 1.850 × height in cm) – (4.676 × age in years)).
(B) Energy expenditure of activities (EEA) was obtained by multiplying REE with physical activity factor of the respondents’ physical activity level (PAL). PAL was determined with WHO global physical activity questionnaire that provided detailed report of types, intensity, frequency and duration (in minutes) of all physical activities (exercise and non-exercise) performed daily for three (3) consecutive days by the respondents [14, 20]. Total physical activity was calculated by summing all the minutes spent on each physical activity category and categorised accordingly into sedentary/light (less than 30 min a day), moderately active (regularly active or accumulated ≥ 30 min per day) and vigorously active (greater intensity activity in ≥ 8–10 min’ bouts in a day) [21]. Physical activity level factor of 1.4 for sedentary/light activity, 1.70 for moderately active, 2.0 for vigorously active were used to account for individual energy expenditure of activity [22, 23]. Energy expenditure of activity (EEA) = Activity factor × REE (kcal/day). Mean of the three days’ values was used in statistical analysis.
(C) Diet-induced energy expenditure was calculated as 10% of total calories consumed in a day [4, 22]. Mean of the three days’ values was used in statistical analysis.

Mean of three days’ energy expenditure was subtracted from the mean of three days’ energy intake to obtain energy balance and interpreted thus: energy intake > energy expenditure = positive energy balance; energy intake < energy expenditure = negative energy balance; energy intake = energy expenditure = equilibrium (energy balance).

### Outcome and predictor variables

The binary outcome variable is energy balance (positive or negative) whereas the exposure variables (covariates) were socioeconomic (age, sex, education, occupation, marital status and income), dietary (skipping meals, number of meals consumed in a day, weekly snack consumption and eating outside the home), lifestyle variables (alcohol consumption, smoking of cigarette/substances), body mass index and waist circumference. Relationships between the outcome and exposure variables were assessed at both the binary and multivariate logistic regression. After examining the individual effects of the above 14 exposure variables at the binary level, they were entered simultaneously into the multivariate logistic model to evaluate the effect of each of the covariates on the outcome variable when other covariates are held constant. Crude and adjusted odds ratios were reported for each of the covariate evaluated.

### Statistical analysis

Data collected were entered into Microsoft excel, validated, cleaned and sorted before being transported into IBM Statistical Product and Service Solutions (version 21) computer software for descriptive and inferential statistical analysis. Descriptive statistics (frequencies and percentages) was used for general characteristics, anthropometric and physical activity levels of the adults. Chi square test was used to evaluate the relationship between categorical variables (anthropometric parameters and physical activity level of the respondents by age and sex as well as the relationship of these parameters with energy intake, expenditure and balance). Means and standard deviations were used for energy intake, expenditure and balance. T-test was used to assess relationships between energy intake, expenditure and balance, and sex, waist circumference and waist hip ratio. Whereas analysis of variance was used to compare the energy parameters among four age groups of the adults and assess the relationship of mean energy intake, expenditure and balance with anthropometric parameters and physical activity level. Binary logistic regression analysis was employed to evaluate associations between the outcome variable and the predictor variables. Since binary logistic regression analysis does not control confounding effects, multivariate logistic regression analysis was conducted to correct for simultaneous effects of multiple factors and control the effects of confounding variables on the response variable. The adjusted odds ratios were used to define the independent strength of the associations. Significance was accepted at 95% precision (*P* < 0.05).

## Results

Mean age (years) of the respondents was 27.7 ± 5.586 (male: 27.7 ± 5.528, female: 27.8 ± 5.658), mean body mass index kg/m^2^ was 25.6 ± 5.156 (male: 25.0 ± 4.379, female: 26.2 ± 5.705), mean waist circumference (cm) was 83.99 ± 13.778 (male: 83.2 ± 11.375, female: 84.7 ± 15.591) and mean waist hip ratio was 0.85 ± 0.071 (male: 0.86 ± 0.060, female: 0.84 ± 0.078).

Table 1 presents the general characteristics of the respondents. More than half (53.3%) of the respondents were females while 46.7% were males. About thirty-five percent (35.4%) of the respondents were within 20 and 24 years while 15.4% were aged 35–39 years. Secondary and tertiary education were attained by 42.9% and 46.3%, respectively. Majority of the respondents were engaged in an occupation (70.8%), never married (56.7%) and among those who were engaged in an occupation, 36.7% earned above ₦50,000 monthly. Most of the respondents (55.8%) consumed three meals daily while only 9.6% consumed more than three meals daily. Majority (69.6%) skipped meals, only 7.1% never consumed snacks and 35.8% never ate outside the home. More than half of the respondents did not consume alcohol (56.2%) and only 15.4% smoked cigarette/substance.

**Table 1:** General characteristics of the respondents

Variables	Frequency	Percentage
**Sex**		
Male	112	46.7
Female	128	53.3
**Age (years)**		
20–24	85	35.4
25–29	71	29.6
30–34	47	19.6
35–39	37	15.4
**Highest educational level attained**		
No formal education	7	2.9
Primary Education	19	7.9
Secondary education	103	42.9
Tertiary education	111	46.3
**Occupation**		
Unengaged	70	29.2
Engaged	170	70.8
**Marital status**		
Ever married	104	43.3
Never married	136	56.7
**Average monthly income (Naira)**		
None	69	28.7
≤ 50,000	83	34.6
> 50, 000	88	36.7
**Number of meals per day**		
One meal	2	0.8
Two meals	81	33.8
Three meals	134	55.8
More than three meals	23	9.6
**Skip meal**		
Yes	167	69.6
No	73	30.4
**Frequency of snack consumption per week**		
Daily	43	17.9
4–6 times/week	28	11.7
1–3 times/week	62	25.8
Occasionally	90	37.5
Never	17	7.1
**Frequency of eating outside the home per week**		
Daily	46	19.2
4–6 times	25	10.4
1–3 times	21	8.8
Occasionally	62	25.8
Never	86	35.8
**Consumption of alcohol**		
Yes	105	43.8
No	135	56.2
Smoking*		
Yes	37	15.4
No	203	84.6

^*^cigarette/substances like cocaine, methamphetamine, Indian hemp

Anthropometric parameters, physical activity level, energy intake, expenditure and balance of the respondents by sex and age are shown in Table 2. There was no significant (*P* > 0.05) difference in body mass index (BMI) of the respondents by sex though prevalence of underweight, overweight and obesity were higher among females. The difference in BMI according to age was significant (*P* < 0.001) with percentage of respondents with normal BMI decreasing as age increases. More females had abdominal obesity than males (*P* < 0.001); prevalence of abdominal obesity increased with increase in age (*P* < 0.05). More females than males had high risk waist hip ratio (*P* < 0.01); the percentage of those with high risk waist hip ratio increased as age increased with those aged 20–24 years having the lowest prevalence and the 35–39 year-olds having the highest; this difference was significant (*P* < 0.001). There was significant difference in physical activity level of the respondents by sex (*P* < 0.001) with more females being vigorously active than males.

**Table 2:** Anthropometric parameters, physical activity level, energy intake, expenditure and balance of the respondents by sex and age

	**Sex**		**Age (years)**				
	**Male**	**Female**	**20–24**	**25–29**	**30–34**	**35–39**	**Total**
**Variables**	**N(%)**	**N(%)**	**N(%)**	**N(%)**	**N(%)**	**N(%)**	**N(%)**
**Body mass index (kg/m**^**2**^**)**							
Underweight (< 18.5)	1(0.9)	2(1.6)	1(1.2)	1(1.4)	1(2.1)	0(0.0)	3(1.3)
Normal (18.5–24.9)	64(57.1)	64(50.0)	59(69.4)	43(60.6)	16(34.0)	10(27.0)	128(53.2)
Overweight (25.0–29.9)	25(22.3)	32(25.0)	13(15.3)	17(23.9)	16(34.0)	11(29.7)	57(23.8)
Obese (≥ 30.0)	22(19.7)	30(23.4)	12(14.1)	10(14.1)	14(29.9)	16(43.3)	52(21.7)
Total	112(100.0)	128(100.0)	85(100.0)	71(100.0)	47(100.0)	37(100.0)	240(100.0)
P value	0.714		0.000***				
**Waist circumference**^**a**^							
Normal	92(82.1)	56(43.8)	62(72.9)	44(62.0)	26(55.3)	16(43.2)	148(61.7)
Abdominal obesity	20(17.9)	72(56.2)	23(27.1)	27(38.0)	21(44.7)	21(56.8)	92(38.3)
Total	112(100.0)	128(100.0)	85(100.0)	71(100.0)	47(100.0)	37(100.0)	240(100.0)
P value	0.000***		0.014*				
**Waist hip ratio (WHR)**^**b**^							
Low risk	81(72.3)	65(50.8)	63(74.1)	44(62.0)	25(53.2)	14(37.8)	146(60.8)
High risk	31(27.7)	63(49.2)	22(25.9)	27(38.0)	22(46.8)	23(62.2)	94(39.2)
Total	112(100.0)	128(100.0)	85(100.0)	71(100.0)	47(100.0)	37(100.0)	240(100.0)
P value	0.001**		0.000***				
**Physical activity level**^**c**^							
Sedentary/lightly active	60(53.6)	36(28.1)	39(45.9)	27(38.0)	16(34.0)	14(37.9)	96(40.0)
Moderately active	43(38.4)	34(26.6)	22(25.9)	23(32.4)	20(42.6)	12(32.4)	77(32.1)
Vigorously active	9(8.0)	58(45.3)	24(28.2)	21(29.6)	11(23.4)	11(29.7)	67(27.9)
Total	112(100.0)	128(100.0)	85(100.0)	71(100.0)	47(100.0)	37(100.0)	240(100.0)
P value	0.000***		0.629				
**Energy balance**							
Positive	69(61.6)	96(75.0)	52(61.2)	45(63.4)	40(85.1)	28(75.7)	165(68.8)
Negative	43(38.4)	32(25.0)	33(38.8)	26(36.6)	7(14.9)	9(24.3)	75(31.2)
Total	112(100.0)	128(100.0)	85(100.0)	71(100.0)	47(100.0)	37(100.0)	240(100.0)
P value	0.026*		0.003**				
**Energy parameters (mean ± SD)**							
Energy intake (kcal/day)	2398.8 ± 494.761	2431.1 ± 876.664	2371.2 ± 1024.695^ab^	2285.3 ± 497.793^a^	2560.7 ± 416.600^ab^	2586.0 ± 467.587^b^	2416.0 ± 722.728
P value	0.731						
Energy expenditure (kcal/day)	2274.3 ± 385.792	2126.6 ± 371.403	2169.4 ± 411.625^a^	2182.7 ± 377.130^a^	2248.8 ± 273.534^a^	2212.6 ± 456.373^a^	2195.5 ± 384.544
P value	0.003**						
Energy balance (kcal/day)	124.5 ± 588.637	304.4 ± 921.041	201.8 ± 1083.158^a^	102.5 ± 629.780^a^	311.9 ± 483.100^a^	373.5 ± 494.546^a^	220.5 ± 787.271
P value	0.077						
% contribution of energy intake to RI^d^	85.7	110.5					

^a^Cut off for abdominal obesity, male > 94 and female > 80 cm

^b^Cut off for high risk WHR, male ≥ 0.90 and female ≥ 0.85 cm

^c^Cut off for daily physical activity, Sedentary/lightly < 30 min; moderately active ≥ 30 min; vigorously active ≥ 8–10 min

^d^FAO/WHO/UNU (2001) daily energy recommendation for males = 2800 kcal; females = 2200 kcal

RI: Recommended intake

SD: Standard deviation

Mean values with different superscripts in the same row are significant at P < 0.05 2-tailed

^*^P < 0.05 **P < 0.01 ***P < 0.001

Males had lower energy intake (kcal/day) (2398.8 ± 494.761) than females (2431.1 ± 876.664) but higher energy expenditure (2274.3 ± 385.792) than females (2126.6 ± 371.403). This relationship was significant (*P* < 0.01) for energy expenditure alone. Female energy intake contributed 110.5% of FAO/WHO/UNU recommended energy intake while male intake contributed less (85.7%). The least energy intake was observed among those aged 25–29 years while the 35–39 year-olds had the highest intake (*P* < 0.05). Energy expenditure and balance among the age groups were similar (P > 0.05). Energy balance was positive among 68.8% of the respondents with a value of 220.5 ± 787.271 kcal/day (124.5 ± 588.637 for males and 304.4 ± 921.041 for females). The least energy balance (kcal/day) was found among the 24–29 year-olds (102.5 ± 629.780) while the 35–39 year-olds had the highest (373.5 ± 494.546). Energy expenditure and balance among the age groups were similar (P > 0.05).

**Fig. 1:**
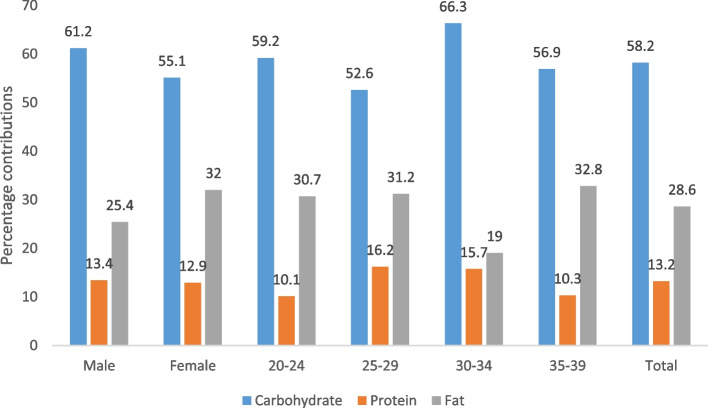
Percentage contributions of carbohydrate, protein and fat to energy intake of the adults by sex and age

Figure 1 shows the percentage contributions of carbohydrate, protein and fat to the energy intakes of the adults by sex and age. Carbohydrate (58.2%) made the highest contribution followed by fat (28.6%) and protein (13.2%). Carbohydrate (61.2%) and protein (13.4%) contributed more in males than females; fat (32.0%) contributed more in females. The 30–34 year-olds had the highest carbohydrate contribution (66.3%), 25–29 had the highest protein (16.2%) while the highest fat (32.8%) contribution was found among the 35–39 year-olds.

Relationship of mean energy intake, expenditure and balance (kcal/day) of respondents with anthropometric parameters and physical activity level are presented in Table 3. Respondents with obesity had the highest energy intake (2733.7 ± 1171.073 kcal/day), expenditure (2431.7 ± 491.666 kcal/day) and balance (302.0 ± 1300.186 kcal/day) while those with underweight had the least (2176.8 ± 222.670, 2031.9 ± 158.939 and 144.8 ± 335.518 kcal/day). Relationship was significant for intake (*P* < 0.01) and expenditure (*P* < 0.001). Those with abdominal obesity had higher energy intake (2573.0 ± 966.553 vs 2318.4 ± 522.192, *P* < 0.01) and expenditure (2226.4 ± 372.621 and 2175.6 ± 392.005, *P* < 0.05) than those with normal waist circumference. Respondents with high risk waist hip ratio also had higher energy intake (kcal/day) of 2567.6 ± 950.366 (*P* < 0.01) and energy expenditure (kcal/day) of 2231.2 ± 396.463 (*P* > 0.05) than those at low risk (2318.4 ± 487.087 and 2170.9 ± 375.538). Energy intake and expenditure were similar across the three physical activity levels (*P* > 0.05) but energy balance was significantly lower among those in the vigorous category and highest in those in the sedentary category (*P* < 0.01).

**Table 3:** Relationship of mean energy intake, expenditure and balance (kcal/day) of respondents with anthropometric parameters and physical activity level

	**Body mass index**				Waist circumference		Waist hip ratio (WHR)		Physical activity level^c^		
Variables	Underweight	Normal	Overweight	Obese	Normal	AO^a^	Low risk	High risk^b^	Sedentary	MA	VA
	Mean ± SD	Mean ± SD	Mean ± SD	Mean ± SD	Mean ± SD	Mean ± SD	Mean ± SD	Mean ± SD	Mean ± SD	Mean ± SD	Mean ± SD
Energy intake	2176.8 ± 222.670^a^	2306.7 ± 522.742^ab^	2384.2 ± 483.417^ab^	2733.7 ± 1171.073^b^	2318.4 ± 522.192	2573.0 ± 966.553	2318.4 ± 487.087	2567.6 ± 950.366	2461.6 ± 938.314^a^	2397.6 ± 475.088^a^	2371.8 ± 599.074^a^
P value	0.003**				0.008**		0.009**		0.566		
Energy expenditure	2031.9 ± 158.939^a^	2106.2 ± 314.785^ab^	2189.3 ± 337.370^ab^	2431.7 ± 491.666^b^	2175.6 ± 392.005	2226.4 ± 372.621	2170.9 ± 375.538	2231.2 ± 396.463	2008.9 ± 329.983^a^	2277.6 ± 330.211^a^	2368.7 ± 405.289^a^
P value	0.000***				0.012*		0.319		0.435		
Energy balance	144.8 ± 335.518^a^	200.5 ± 589.763^a^	194.9 ± 552.066^a^	302.0 ± 1300.186^a^	142.8 ± 544.098	341.2 ± 1052.145	129.1 ± 536.164	352.8 ± 1038.912	425.7 ± 1000.696^c^	120.1 ± 501.534^b^	3.1 ± 612.552^a^
*P* value	0.868				0.224		0.056		0.007**		

*MA* Moderately active, *VA* Vigorously active

Means with similar superscripts in the same row for each variable are not significantly (*P* > 0.05) different at 2 tailed

^a^AO, Abdominal obesity

^a^Cut off for abdominal obesity, male > 94 and female > 80 cm

^b^Cut off for high risk WHR, male ≥ 0.90 and female ≥ 0.85 cm

^c^Cut off for daily physical activity = Sedentary/lightly < 30 min; moderately active ≥ 30 min; vigorously active ≥ 8–10 min

^*^P < 0.05 **P < 0.01 ***P < 0.001

Table 4 shows the factors associated with energy balance of the respondents. Only two variables attained significance (*P<0.05*) in the multivariate model. Respondents less than 30 years had nearly 3 times higher likelihood (AOR: 2.89, 95% C.I.: 1.27–6.56) of having positive energy balance than those within the ages of 30 to 39 years. Those who were not engaged in any occupation were 2 times more likely to have positive energy balance than those who were engaged in an occupation (AOR: 2.30, 95% C.I.: 1.05–5.03). Though not significant, being a male (AOR: 1.94, 95% C.I: 0.93–4.01), eating outside the home (AOR: 1.51, 95% C.I.: 0.75–3.05) and smoking cigarette or any other substance (AOR: 2.05, 95% C.I.: 0.82–5.11) placed the respondents at almost 2 times higher risk of positive energy balance. The likelihood of having positive energy balance decreased as body mass index increased though this did not attain significant proportions.

**Table 4:** Factors associated with positive energy balance of the respondents

**Variables**	**Energy balance**					
	**Positive**	**Negative**	**UOR(95%) C.I**	**P value**	**AOR(95%) C.I**	**P value**
**Sex**						
Female	96(58.2)	32(42.7)				
Male	69(41.8)	43(57.3)	1.87(1.08–3.25)	0.026*	1.94(0.93–4.01)	0.076
**Age**						
≥ 30 years	69(41.8)	16(21.3)				
< 30 years	96(58.2)	59(78.7)	2.65(1.41–4.99)	0.003**	2.89(1.27–6.56)	0.011*
**Highest educational level completed**						
No formal	4(2.4)	3(4.0)				
Primary	13(7.9)	6(8.0)	0.54(0.11–2.55)	0.437	0.43(0.08–2.51)	0.351
Secondary	69(41.8)	34(45.3)	0.88(0.31–3.51)	0.808	1.04(0.33–3.28)	0.943
Tertiary	79(47.9)	32(42.7)	0.82(0.46–1.47)	0.508	1.10(0.55–2.20)	0.788
**Occupation**						
Engaged	127(77.0)	43(57.3)				
Unengaged	38(23.0)	33(42.7)	2.49(1.39–4.46)	0.002**	2.30(1.05–5.03)	0.037*
**Marital status**						
Ever married	74(44.8)	30(40.0)				
Never married	91(55.2)	45(60.0)	1.22(0.70–2.12)	0.483	0.57(0.29–1.15)	0.119
**Average monthly income**						
None- ≤ 50,000.00	88(60.0)	53(70.7)				
> 50,000.00	66(40.0)	22(29.3)	0.43(0.22–0.84)	0.014*	1.05(0.48–2.30)	0.894
**Skipping of meals**						
No	54(32.7)	19(25.3)				
Yes	111(67.3)	56(74.7)	1.43(0.78–2.65)	0.250	1.27(0.60–2.67)	0.533
**Number of meals per day**						
≥ 3	110(66.7)	47(62.7)				
< 3	55(33.3)	28(37.3)	1.19(0.68–2.11)	0.546	0.96(0.49–1.91)	0.912
**Snack consumption per week**						
> 3 times	42(25.5)	29(38.7)				
≤ 3 times	123(74.5)	46(61.3)	0.54(0.30–0.97)	0.039*	0.70(0.36–1.36)	0.290
**Eating outside the home**						
No	65(39.4)	21(28.0)				
Yes	100(60.6)	54(72.0)	1.67(0.92–3.02)	0.090	1.51(0.75–3.05)	0.254
**Alcohol consumption**						
No	92(55.8)	43(57.3)				
Yes	73(44.2)	32(42.7)	0.94(0.54–1.63)	0.820	0.59(0.29–1.20)	0.145
**Smoking substances**^**a**^						
No	144(87.3)	59(78.7)				
Yes	21(12.7)	16(21.3)	1.86(0.91–3.81)	0.090	2.05(0.82–5.11)	0.124
**Body mass index (kg/m**^**2**^**)**						
Underweight (< 18.5)	2(1.2)	1(1.3)				
Normal (18.5–24.9)	89(53.9)	39(52.0)	1.06(0.09–12.49)	0.964	3.00(0.18–49.86)	0.444
Overweight (25.0–29.9)	40(24.2)	17(22.7)	1.21(0.61–2.40)	0.588	2.48(0.91–6.74)	0.076
Obese (≥ 30.0)	34(20.7)	18(24.0)	1.25(0.56–2.79)	0.593	1.69(0.63–4.50)	0.297
**Abdominal obesity**						
Absent	99(60.0)	49(65.3)				
Present	66(40.0)	26(34.7)	1.26(0.71–2.22)	0.431	1.15(0.48–2.73)	0.756

Positive (energy intake > energy expenditure) *N* = 165(68.8%)

Negative (energy intake < energy expenditure) *N* = 75(31.2%)

*UOR* Unadjusted odds ratio, *AOR* Adjusted odds ratio

^a^Substances like cocaine, methamphetamine, Indian hemp

^*^P < 0.05

## Discussion

This study which assessed the energy intake, energy expenditure and energy balance of young adults (20–39 years) and examined factors associated with their energy balance was conducted in southeast Nigerian urban setting.

The study revealed a mean female energy intake similar to 2428 kcal/day reported among female pre-professional dancers [24] and 2327.0 kcal/day among Jamaican African females [25]. While Hattingh et al. [26] reported a lower value of 2969.7 kcal/day (12,425.4 kJ), Fyfe et al. [27] reported a higher of 1988.8 (8321 kJ). The mean energy intake of males reported in this study is similar to 2497.6 kcal (10,450 kJ) reported by Fyfe et al. [27]. In comparison with FAO/WHO/UNU recommendations [23], female intake is higher than 2,200 kilocalories required daily by a healthy adult female and male intake is lower than the 2,800 kilocalories recommended daily for a healthy adult male. Fyfe et al. [27] also reported that female energy intake was 1% higher than value recommended for them while male energy intake was 5% lower the recommended amount. According to Bennette et al. [28], though men had greater energy intake, they were less likely to have energy intakes above the estimated average requirement compared to women. This is in line with the findings of this study in which male intake contributed only 85.7% of the recommended daily energy requirement. This means that other nutrient requirements will also not be met because all other nutrients must be provided within the quantity of food required to fulfil the energy requirements [29]. Energy intake reported in this study may be functions of portion sizes and diet composition. Fatty foods and diets contribute more to energy intake than carbohydrate and protein. We observed that fat intake contributed more than 30% of the total energy intake among respondents below 30 years and above 34 years. A study also reported a higher percentage contribution from fat and less from carbohydrate and protein to energy intake [24]. According to Sudo et al. [30], larger food portion sizes resulted in larger daily energy intake per body weight. Females have been reported to consume foods more times during the day and uncontrollably too [31]; this may be responsible for the higher energy intake observed among them though relationship with male intake was not significant.

This study also showed that the mean daily total energy expenditure of the females was lower than 2784 kcal/day reported among female pre professional dancers by Brown et al*. *[24]. Mean energy expenditure of the males was significantly higher than that of the females. This is in line with the report of Redman et al. [32] who reported that daily energy expenditure of 2443 kcal/day among males was 20% higher than in females. This higher energy expenditure in males could be attributed to larger muscle mass.

Contrary to the findings of other researchers [24, 27] on energy balance, this study reported positive energy balance among males and females raising concerns over weight gain if sustained. Very small differences have been shown to lead to important gains in weight over time [33]. The positive energy balance of most of the respondents in this study may have contributed to the high prevalence of overweight and obesity among them. The higher mean energy balance among females implies possible weight gain in the face of low energy expenditure. That up to 37.9% of the respondents were sedentary/lightly active is a cause for worry and calls for strategies to increase physical activity.

Multivariate logistic regression analysis showed that respondents who were less than 30 years were more likely to have positive energy balance than those aged 30 years and above. This may be a consequence of consumption of energy dense foods and beverages coupled with newly gained socioeconomic independence to make food choices. Livingstone et al. [34] found a strong relationship between consumption of energy dense dietary pattern high in free sugars and saturated fatty acids and obesity among young adults.

The likelihood of having positive energy balance increased by 2 among those who were not engaged in any occupation. This was attributed to low physical activity. Being engaged in an occupation increases energy expenditure though research has shown reduction in occupation related energy expenditure and reported that increases observed in fat percentage and body mass index are independent of occupation [35, 36]. Those not engaged in any occupation do not benefit from any occupation related activity and therefore, more likely to have low physical activity level which leads to positive energy balance, sustained weight gains and consequences of obesity.

Though not significant, respondents who eat outside their homes were almost 2 times more likely to have positive energy balance. Most foods consumed outside homes are fast foods and fast food consumers have been reported to have higher mean energy, carbohydrate, protein and fat intakes than non-fast food consumers [37]. Fast foods are mainly energy dense nutrient-poor foods and beverages. It was not a surprise therefore that three or less times weekly snack consumption was associated with less likelihood of having positive energy balance than a higher consumption of above three times a week; though this is not significant.

Smoking of cigarette and other substances increased the risk of having positive energy balance by 2; this however did not reach significant proportions. In affirmation, a strong linear relationship was observed between smoking pattern and dietary energy density in current smokers with daily and non-daily smokers having significantly higher dietary energy density than non-smokers [38]. In a study to determine the effect of smoking status on total energy expenditure, the authors [39] reported no significant differences in total energy expenditure between smokers and non-smokers implying that the issue may lie with energy intake. That smoking significantly reduced dietary calorie intake [40] was contrary to our findings and may be attributed to type, frequency and quantity of smoke inhaled.

Interestingly, the likelihood of having positive energy balance decreased as body mass index increased showing that those with normal body mass index were more likely to have positive energy balance than those with overweight and obesity. This may be attributed to lack of caution in consuming energy dense foods and drinks. People with normal BMI should, therefore, guard against excessive energy intake and low physical activity level as it may lead to weight gain and retention.

### Limitations

This study is not without limitations. Firstly, the study was limited to an urban area in southeast Nigeria which did not represent the whole of Nigeria. Secondly, we did not use the doubly labelled water method which is the gold standard method to assess body metabolic rate and body composition of the adults was not assessed. Thirdly, the study employed self-reported retrospective data on food intake and physical activity which may be associated with recall bias. Fourthly, portion sizes on which the energy intake was based was not presented. Lastly, in assessing the factors associated with energy balance, cause and effect associations could not be established through a cross sectional study.

## Conclusion

This study showed higher female daily energy intake than male intake with lower daily energy expenditure than males. The overall energy balance was positive. Age and occupation contributed to positive energy balance among the respondents. These findings are vital to planning nutrition and health education, and dietetic management of individuals prone to obesity.

## Data Availability

Data generated from this study on which the results are based are available from the corresponding authors on reasonable request.

## Abbreviations

TEI: Total energy intake
EI: Energy intake
EE: Energy expenditure
TEE: Total energy expenditure
REE: Resting energy expenditure
EEA: Energy expenditure of activity
DEE: Diet-induced energy expenditure
UOR: Unadjusted odds ratio
AOR: Adjusted odds ratio
C.I.: Confidence interval
